# Progresses in Wound Healing: Integrating Nutrition, Physical Therapy, Traditional and Alternative Medicine, and Novel Technologies

**DOI:** 10.2174/0115733998350164250228174809

**Published:** 2025-04-10

**Authors:** Phool Chandra, Shahjad, Mayur Porwal, Vaibhav Rastogi, Piyush Mittal, Anurag Verma, Neetu Sachan, Arulsamy Elphine Prabahar, Manish Kumar Gautam

**Affiliations:** 1 Teerthanker Mahaveer College of Pharmacy, Teerthanker Mahaveer University, Moradabad, U.P., 244001, India;; 2 Department of Pharmacology, Global Institute of Pharmaceutical Education and Research, Kashipur, 244713, India;; 3 Maharana Pratap College of Pharmacy, Mandhana, Kanpur, Moradabad, U.P., India;; 4 Chettinad School of Pharmaceutical Sciences, Chettinad Hospital and Research Institute, Chettinad Academy of Research and Education, Kelambakkam, 603103, Tamil Nadu, India;; 5 Faculty of Pharmaceutical Science, Assam down town University, Sankar Madhab Path, Gandhi Nagar, Panikhaiti, Guwahati, 781026, Assam, India

**Keywords:** Traditional medicine, alternative medicine, novel strategies, wound healing, nanofibers, chronic wounds

## Abstract

Wound healing is a complex, tightly regulated biological process essential for restoring the integrity and functionality of damaged skin. Chronic wounds, affecting approximately 6.5 million individuals globally and 1.5% of the Indian population, pose significant healthcare challenges due to their prolonged and intricate healing processes. This review highlights the mechanisms and stages of wound healing—hemostasis, inflammation, proliferation, and maturation—emphasizing the bidirectional influence of internal and external factors on wound progression. Despite advancements in wound management, current therapeutic options, including skin grafts, growth factors, and cell-based therapies, often remain inadequate for diverse wound types. Emerging technologies, such as nanofibers, stem cell therapy, 3D bioprinting, fluorescence imaging, and bacteria-killing laser therapy, offer promising solutions by enhancing therapeutic outcomes and minimizing side effects. This review also explores the roles of nutrition, physical therapy, and traditional medicine in promoting effective wound care. By integrating novel technologies with established therapeutic strategies, this work provides a comprehensive overview of advanced wound healing modalities, their mechanisms, advantages, and limitations. The review concludes with a perspective on future research directions aimed at overcoming current challenges in chronic wound management and optimizing patient outcomes.

## 1 INTRODUCTION

Wound healing is the body's common restorative technique for repairing damaged tissues [1]. Depending on how severe the damage is, it is a dynamic and carefully planned procedure that starts as soon as it happens and can take days or weeks to complete [2]. The etiology of wound healing is typically categorized according to the three phases of repair: hemostasis, inflammation, and remodeling [3]. Damaged blood arteries contract and platelets coagulate to stop bleeding during the hemostasis phase. During the inflammatory phase, immune cells and various chemicals are recruited to the site to promote healing by removing debris, promoting new cell growth, and assisting tissue creation. Collagen and other support are used throughout the rebuilding phase. The time it takes for a wound to heal might vary depending on its type, from a few days to months [4]. Wounds are categorized as either chronic or acute. Acute wounds are caused by trauma and typically heal in three to four weeks, while chronic wounds persist for long periods, either due to underlying medical conditions or inadequate care. Chronic wounds represent a significant global health burden, affecting an estimated 6.5 million individuals worldwide. In the United States, approximately 10% of the population suffers from chronic wounds, with conditions such as diabetes and venous insufficiency contributing to the prevalence [5]. Approximately 6 million people worldwide are affected by chronic, non-healing wounds, underscoring the disparity in healthcare outcomes [6]. These wounds often lead to prolonged morbidity, increased healthcare costs, and diminished quality of life. Despite advances in wound management, the intricate interplay of cellular and molecular processes in wound healing presents ongoing challenges. Addressing these issues requires a multifaceted approach integrating traditional therapies with cutting-edge technologies, as discussed in this review. Common causes for these wounds are diabetes, venous insufficiency, and pressure ulcers. Poor nutrition, obesity, smoking, and inadequate wound care can all contribute to a longer healing process [7]. The prevalence of chronic wounds varies from state to state in India. According to one study, West Bengal is the state with the highest number of individuals having chronic wounds, followed by Kerala, Maharashtra, and Tamil Nadu. Other than those four states, the study found that Uttar Pradesh, Gujarat, Andhra Pradesh, Karnataka, Madhya Pradesh, Rajasthan, Bihar, Delhi, Punjab, Haryana, and Orissa also have high prevalence rates of chronic Abrasions [8]. Types of wounds are presented in Fig. (**1**).

### 1.1 Abrasions

Abrasion wounds result from the impact of blunt or sharp objects scraping the skin, leading to superficial or deep injuries. These wounds are the initial stage of tissue disruption and typically require cleaning to prevent infection, with more severe cases necessitating antibiotics or sutures [9]. Immediate cleaning, antibiotic application, and monitoring are critical for these wounds [10].

### 1.2 Lacerations

A laceration is a tear or cut in the skin and is typically caused by blunt force trauma and may range from superficial to deep [10]. Treatment depends on the location and severity of the wound but may comprise cleaning the wound, suturing, antibiotics, and pain medications treatment for some lacerations may include wound irrigation, wound closure with stitches or glue, cleaning the wound, and/or antibiotics [11]. Pain medications may be additionally prescribed to help make the person more comfortable during the healing process.

### 1.3 Punctures

Puncture wounds are characterized by small, round perforations in the skin. Proper care is essential to prevent infection. The wound should be thoroughly cleansed with soap and water, followed by the application of an antibiotic cream or ointment. Subsequently, the wound should be covered with a sterile bandage or dressing. If the wound exhibits delayed healing or signs of infection, medical attention should be promptly sought. Adhering to these care practices minimizes the risk of complications and promotes effective recovery [12].

### 1.4 Avulsions

An avulsion wound is a severe type of laceration where tissues are forcibly separated from the body due to a violent external force. This may result in the detachment of skin or deeper tissues. Such injuries can occur from blunt trauma, sharp objects, or extreme pressure [13]. Typically, stitches are needed to seal an avulsion incision and avoid infection. For the treatment of avulsion wounds, it is important to clean the wound and stop any bleeding. This can be done by applying pressure directly to the wound with a clean cloth or gauze [14]. A sterile bandage and medical tape should be applied to the wound after the bleeding has ceased. Antibiotics may be prescribed to prevent infection and reduce healing time. Surgery can be required to repair the tissue, depending on how serious the injury is. The use of ice or a cold compress can help to reduce swelling and pain [15]. It is important to keep the wound clean and dry and avoid strenuous activity until it has healed. It is also important to monitor the wound regularly and change the bandage as needed. Before placing a new bandage, the region must be washed with warm water and mild soap. Whenever possible, keep the wound elevated above the level of the heart to minimize swelling and inflammation. Over-the-counter pain relievers can help alleviate discomfort. Need consultation if signs of infection, such as redness, increased swelling, fever, or the presence of pus. Additionally, ensure adequate rest and maintain a balanced diet to support healing and recovery [16].

### 1.5 Incisions

An incision is a surgical cut made in the skin of a person or animal, usually to perform a procedure or gain access to an internal organ. An incision is a wound caused by cutting into the skin or other body tissue. It is typically done with a scalpel or razor blade and is an essential part of many surgical procedures. Incision wounds are usually very precise and often help to reduce the amount of bleeding associated with a procedure as well as decrease the risk of tissue damage or infection. Incision wounds are left to heal on their own unless a foreign object needs to be removed or stitches are needed to close the wound. After healing, a scar may remain at the site of the incision [17].

### 1.6 Contusions

Contusions, commonly known as bruises, result from trauma to the skin and underlying tissues. They are characterized by swelling, discoloration (typically purple or blue), and tenderness. Contusions are most often caused by blunt force, such as a punch, fall, or impact with an object [18]. The trauma leads to bleeding under the skin, causing swelling and discoloration. Treatment typically includes rest, ice application, and elevation of the affected area to reduce swelling, and pain relief medications. In severe cases, physical therapy may be recommended to aid recovery [19].

### 1.7 Burns

Burn wounds are the result of contact with extreme heat, chemicals, electricity, radiation, or friction, and can cause serious damage to the skin and underlying tissue. Treatment options may include topical ointments and dressings, surgery, and skin grafts [20].

### 1.8 Ulcers

Ulcers are open sores that can appear on the skin or mucous membranes, such as those in the mouth or stomach. They are often painful and can last for days, weeks, or months at a time. Ulcer wounds can be caused by an infection or an underlying medical condition, such as diabetes. Treatment for ulcers depends on the cause and may include antibiotics, antacids, anti-inflammatory medications, or diet changes [21].

## 2 FACTORS AFFECTING WOUND HEALING

Factors affecting the wounds are presented in the Fig. (**2**).

### 2.1 Internal Factors

#### 2.1.1 Age

Age is a significant factor influencing wound healing, as it impacts various physiological processes essential for tissue repair. Poor nutrition, often associated with aging, can impair the body’s ability to produce collagen—a crucial protein for wound healing. This deficiency slows tissue regeneration and weakens the skin’s structural integrity. Additionally, inadequate nutrition compromises the immune system, reducing the body’s ability to combat infections and increasing the risk of delayed healing or wound infections. Poor dietary intake can also affect the body’s inflammatory response, potentially leading to prolonged inflammation, increased pain, and swelling. Moreover, malnutrition can result in anaemia, which further delays healing by limiting oxygen delivery to the wound site, a critical factor for tissue repair [22]. Addressing nutritional deficiencies is essential for optimizing wound healing, especially in older adults.

#### 2.1.2 Health Status

Wound healing can be adversely affected by a person's state of health. Poor nutrition, lack of exercise, smoking, and other unhealthy lifestyle choices can all contribute to impaired wound healing. Poor health can also increase the risk of infection, further delaying wound healing. Additionally, certain chronic medical conditions, such as diabetes, can also impair wound healing [23].

#### 2.1.3 Body Fluid

Body fluids can affect wound healing in a variety of ways. For example, excessive fluid loss from a wound can lead to dehydration, slowing down the healing process. Additionally, the presence of bacteria or other contaminants in the body fluid can lead to infection, which can also impede wound healing. Finally, Body fluids may alter the pH balance of a wound, which may slow down healing [23].

#### 2.1.4 Nutritional Status

Nutrition is crucial for the healing of wounds. Adequate nutrition is essential for the body to produce the energy and protein necessary for tissue repair. Nutritional deficits may result in slowed or impaired wound healing. The body's capacity to fight infection, produce collagen, and create new blood vessels can all be negatively impacted by malnutrition. Nutrients such as protein, zinc, vitamin C, and vitamin A are crucial for the healing of wounds [23]. Protein is essential for the synthesis of collagen, a key element of the extracellular matrix and a critical factor in wound healing. Zinc is included in the formation of new tissue, and vitamin C is necessary for the production of collagen. The creation of new blood vessels, which is crucial for wound healing, depends on vitamin A. Therefore, it is important to maintain adequate nutrition to ensure proper wound healing [24].

### 2.2 External Factors

#### 2.2.1 Infection

Wound infection can be defined as the invasion of a wound site by disease-causing microorganisms, leading to local tissue damage and potential systemic illness. Factors that can contribute to wound infection include prolonged exposure to moisture and oxygen, the presence of debris in the wound, wound age, type of bacteria present, immune status of the patient, use of antibiotics, and wound location. It is important to remember that even with the right wound care, infection of the wound can still occur, Therefore, it's critical to be aware of the symptoms and signs of infection and seek medical care as necessary [25].

#### 2.2.2 Medication

Medications play a vital role in wound healing by addressing underlying infections, reducing inflammation, and managing pain. However, wound care involves more than just medication. Essential factors include preventing further injury, maintaining cleanliness and dryness of the wound, and protecting it from external contaminants like dirt and debris. Proper nutrition, adequate rest, and regular exercise also significantly contribute to speeding up the healing process. Additionally, following medical advice on regular wound dressing changes is crucial, and seeking professional help is necessary if the wound does not show improvement within a reasonable time [25].

#### 2.2.3 Temperature

Among the most critical elements impacting wound healing is temperature. Wounds heal more quickly in warm temperatures, as heat increases blood flow to the affected area and helps activate the body's immune system. Cold temperatures, on the other hand, can slow down wound healing by reducing blood circulation to the wound area and resulting in blood vessel constriction. Additionally, cold temperatures can also increase inflammation, leading to further complications in healing [26].

#### 2.2.4 Stress

A range of stressors can have an impact on wounds. These include physical or environmental stresses, such as extreme temperatures, high humidity, direct sunlight, and other forms of exposure; biological stresses, such as infection, systemic illness, allergies, and immune system dysfunction; nutritional deficiencies; and psychological stresses, such as anxiety, depression, and trauma. The severity of a wound can be affected and even worsened by any of these stress factors, and it is important to keep them in mind when assessing and treating a wound [27].

#### 2.2.5 Desecration

The various factors that can affect wound healing include physical activity, nutrition, chronic illness, age, and the type of wound itself. Physical activity can slow down wound healing, especially in the case of high-impact activities. Inadequate nutrition can also increase the risk of infection and hinder the healing of wounds [28]. Chronic illnesses, such as diabetes or cancer, may also impede wound healing. Age is also a factor, as elderly individuals often experience slower wound healing due to reduced skin elasticity and circulation. The type of wound itself can further influence the rate of healing, as some wounds, such as burn wounds, may require advanced medical treatment and extended time to heal [29].

## 3 THE ROLE OF NUTRITION IN WOUND HEALING

Wound healing is significantly influenced by nutrition. Nutrients provide the fuel necessary to produce cells and keep them healthy. They also provide the building blocks for new tissue, such as collagen and epithelial cells [30]. Protein is crucialas it helps to repair and rebuild damaged tissues. The vitamins A, C, and E, as well as the minerals zinc and iron, are additional vital nutrients. Proper nutrition is key to successful wound healing because it supplies the body with the necessary energy to fight infection and produce new cells [31]. It is crucial to have a balanced diet that includes fruits, vegetables, whole grains, proteins, and healthy fats to give the body the nutrition it needs to repair wounds. Drinking enough water is also essential for successful healing as it helps to flush out toxins Humans also need to take adequate rest and reduce stress levels during the wound healing process [32]. Getting enough sleep and managing stress through relaxation techniques can help to boost the body's healing process. Additionally, avoiding smoking and excessive alcohol consumption can also improve wound healing. With proper nutrition and lifestyle modifications, wounds can heal faster and with fewer complications [33]. It is important for individuals who have open wounds to take extra care, as their risk of infection is increased. Keeping the wound clean and following proper sterilization techniques can help prevent infection and shorten the duration of the wound's healing [34]. Additionally, individuals should keep their wounds covered with a sterile dressing to protect the area from bacterial contamination. Ultimately, proper nutrition and taking the recommended steps to care for a wound can help to speed up the healing process. Eating a balanced diet, getting enough rest, and following the advice of a doctor or healthcare professional can all go a long way in helping to ensure successful wound healing [35].

## 4 THE ROLE OF PHYSICAL THERAPY IN WOUND HEALING

Physical therapy plays an important role in wound healing. Physical therapists provide specialized treatments to promote healing, including therapeutic exercises, massage, stretching, and joint mobilizations [36]. They also provide strategies for controlling pain and swelling, as well as helping to prevent complications such as infection and scarring. Physical therapy can improve circulation and range of motion, all of which can help facilitate the healing process, reduce scarring, and reduce the risk of infection [37]. Physical therapists can also advise on wound care, rehabilitation, and return to normal activities. Physical therapists also provide education and support to individuals with chronic wounds [38]. They provide advice on the types of dressings to use and how to properly care for the wound, advice on proper nutrition to promote healing, and help with lifestyle modifications that can improve overall health and well-being. Physical therapy can also help individuals regain functionality and strength, as well as help some individuals return to pre-injury activities [39].

### 4.1 Therapeutic Exercises

The healing of wounds can benefit greatly from therapeutic exercise, especially in the early stages of recovery. Exercises aid in boosting blood flow to an affected area, which provides more oxygen and nutrients to the wound, aiding in the healing process. Additionally, Exercise can improve muscle strength, flexibility, and joint range of motion while reducing swelling and inflammation. These benefits can improve the overall healing process, reducing the risk of infection and prolonging the healing time [40].

### 4.2 Massage

In the past, people have employed massage treatment to aid in the healing of wounds. Massage helps to reduce inflammation, increase circulation, and speed up the healing process. It is also known to help reduce pain, improve range of motion and function, reduce stress, and promote relaxation [41]. Massage therapy can be used to apply light pressure to the wound area, to encourage proper alignment of scar tissue, to break down adhesions and abnormal fibrous tissue, and to reduce edema. This can not only accelerate wound healing but also improve the result in terms of aesthetics and functionality [42].

### 4.3 Stretching

Stretching can aid in the healing of wounds by enhancing circulation, reduce inflammation and swelling, reducing strain on the affected area, and strengthening the affected muscles. Stretching can help the body heal quicker and more thoroughly, allowing for improved movement and faster recovery. Stretching also helps to prevent scarring and can help promote proper tissue regeneration. Proper stretching during the healing process can help reduce scarring and improve function [43].

### 4.4 Joint Mobilizations

Joint mobilizations are beneficial in the wound healing process by improving the range of motion, reducing swelling, and minimizing scar tissue formation around the wound [44]. They also help alleviate pain, enhance blood flow, and promote circulation, all of which are critical for healing. Additionally, joint mobilizations can reduce the risk of complications like infection and adhesions that may slow recovery. By improving circulation, they facilitate the delivery of nutrients and healing components to the affected area [45].

## 5 THE ROLE OF TRADITIONAL MEDICINE IN WOUND HEALING

Traditional medicine has been used for wound healing since ancient times. Various botanical drugs in traditional medicine including Chinese medicine have been used for the treatment of wounds since ancient times. Nowadays, multiple wound-healing therapeutics derived from botanical drugs are commercially available worldwide. Among all the botanical drugs in traditional medicine reported to possess wound healing activities, several drugs havebeen extensively investigated and clinically proven to be effective, such as aloe vera, honey, turmeric, and coconut oil (Table **1**).

### 5.1 Aloe Vera

Aloe vera offers wound-healing qualities that could hasten skin regeneration. It has potent antibacterial and anti-inflammatory qualities that can help lessen pain, swelling, and redness. It helps keep the skin moist and provides a protective barrier against dirt and bacteria that can cause infections [72]. Aloe vera gel also contains many vitamins and minerals that provide nourishment to the wound, aiding in its healing. Aloe vera has also been demonstrated to have antioxidant capabilities, which can help protect the skin from further damage due to free radical activity [73]. It also helps to stimulate collagen production, which helps to promote skin cell regeneration and strengthens the skin to aid in wound healing. Aloe vera may also help speed up the process of healing wounds and scars by promoting new skin growth [74]. Aloe vera gel applied to a wound can help reduce inflammation and itching, while also helping to moisturize the skin and promote proper healing. Additionally, aloe vera possesses astringent properties that aid in drying out wounds, promoting faster healing [75]. Aloe vera may also help promote wound healing when taken orally. It has been found to help support the immune system, which helps to fight off any infections that may arise during the healing process. Aloe vera aids in minimizing scarring by reducing inflammation and promoting healthy cell regeneration. Also, aloe vera has antifungal properties which can help to prevent fungal infections from occurring in a wound [76].

### 5.2 Honey

Honey's antibacterial and anti-inflammatory qualities have been utilized to promote wound healing for millennia. Studies have shown that when honey is applied to wounds, it helps reduce swelling, redness, and pain, as well as speeds up the healing process. Honey also helps keep wounds clean and free from infection by creating an environment that is hostile to bacteria [77]. In addition, honey has a low pH which makes it difficult for bacteria to flourish. Honey also has antioxidant properties, which can help reduce oxidative stress from free radicals and support the overall healing process. Honey is a natural humectant, meaning it helps to keep the wound moist which is essential for healthy healing [78]. Additionally, honey contains healing enzymes that help break down and clear away dead tissue, while also promoting new tissue growth. Burns have traditionally been treated with honey, cuts, and abrasions due to theirability to help reduce wound size and promote healing [79]. Additionally, honey can be used as a topical treatment for skin irritations such as eczema, dermatitis, and psoriasis, as well as to help treat fungal infections. Studies also show that honey can help reduce scarring by aiding in the formation of new skin cells, as well as helping to minimize redness and discoloration [80]. Honey can also be beneficial for wound pain management, providing a soothing and cooling effect on the skin that helps relieve inflammation and discomfort. It is crucial to keep in mind that some kinds of honey are more suitable for wound treatment than others [81]. Manuka honey is a popular choice due to its additional anti-bacterial and immune-boosting properties. Additionally, raw or unpasteurized honey is preferable as it retains more of the healing enzymes [82]. When using honey for wound care, it is important to clean the wound first before applying a small amount of honey directly to the wound. Ensure that the honey is not too hot as this could cause discomfort. Cover the wound with a sterile dressing and change it regularly to keep the wound clean and moist. Honey can also be mixed with other natural ingredients such as olive oil or shea butter to create a healing ointment [83].

### 5.3 Turmeric

Turmeric is renowned for its potent antioxidant and anti-inflammatory qualities. which makes it very beneficial for wound healing. It can help in reducing pain and swelling at the site of the wound, as well as speed up the process of healing [84]. Turmeric also helps to prevent infection by fighting off bacteria and fungi, and can also help to reduce bruising and scarring. Boils, eczema, and psoriasis are just a few of the skin problems that have been successfully treated with turmeric. Additionally, it has been shown to help in the treatment of burns and cuts, as well as reduce the appearance of scars [85]. Additionally, turmeric can help to reduce redness, itching, and swelling associated with skin wounds and irritations. Finally, turmeric can also help to boost the immune system and promote collagen formation, which can help to strengthen skin tissue, reducing the risk of future damage and infections [86]. Although, turmeric might speed up the healing of wounds, it is not a substitute for professional medical care. It is also important to take caution when using turmeric on open wounds and to talk to a healthcare provider if skin irritation occurs [87]. Turmeric is a safe and natural way to help with the healing of wounds, but it should be used under the guidance of a healthcare provider. In addition to its capacity to heal wounds, turmeric has a variety of other health benefits. It is thought to help with inflammation, digestion, and even weight management. Turmeric is also believed to have antioxidant and antimicrobial properties, which could aid in lowering the risk of infection and sickness [88].

### 5.4 Tea Tree Oil

Tea tree oil has been used historically by the Aboriginal people of Australia for wound healing and other health and beauty concerns. More recently, scientific studies have started to validate the effectiveness of this traditional remedy. The antibacterial, antifungal, antiviral, and anti-inflammatory effects of tea tree oil are well known, and research has shown that it may help promote wound healing in numerous ways [89]. It can promote the growth of skin cells at the wound site, lessen inflammation, and eliminate germs and fungi. It is also believed the healing process more quickly, reduces pain, swelling, and irritation, and reduces the risk of infection [90]. Tea tree oil is often used in combination with conventional medical treatments such as antibiotics, bandages, and dressings, and is widely available as a gel, cream, lotion, or ointment. Tea tree oil can also be used in the form of a compress, diluted in water, or added to baths or compresses [91]. However, it is important to be aware that although tea tree oil is generally considered safe when used topically, some people may experience sensitivity and skin irritation. It should also not be ingested or applied to deep or open wounds [92]. Finally, it is crucial to keep in mind that tea tree oil shouldn't be used in place of medical attention; instead, if a wound does not heal properly, you should consult a doctor or other healthcare expert. It can also be beneficial to consult with a naturopathic practitioner or aromatherapist for further advice regarding the safe application of tea tree oil for wound healing [93].

### 5.5 Coconut Oil

Coconut oil has been studied for its potential role in wound healing. According to research, the antibacterial and antimicrobial properties of coconut oil may help protect against infection, reduce inflammation, and promote healing. In addition, the fatty acids in coconut oil can help keep the skin moisturized and promote elasticity, which can lead to faster healing [94]. Coconut oil may also help stimulate collagen synthesis, which can lead to stronger, healthier skin cells. Therefore, while more research is needed, coconut oil may be a beneficial supplement to traditional methods of wound healing. Additionally, coconut oil may be used to promote the growth of new tissue [95]. Coconut oil contains lauric acid, which is thought to promote cell repair and regeneration. This can result in faster wound healing and reduced scarring. Furthermore, studies have shown that when applied topically, coconut oil can help reduce inflammation and pain associated with wounds [96]. A small layer of coconut oil applied to the affected region may also be beneficial lock in moisture and protecting the wound from further contamination. Finally, coconut oil can also be a great way to cleanse and protect the wound. Coconut oil contains medium-chain triglycerides (MCTs) that act as an effective and gentle cleanser. MCTs possess antibacterial, antifungal, and antiviral properties that may help lower the risk of infection. Applying a small amount of coconut oil to the wound can help keep it clean by preventing dirt, debris, and bacteria buildup, supporting the healing process [97].

### 5.6 Sesame Oil

The use of sesame oil for wound healing stretches back thousands of years. In traditional Chinese medicine and Ayurvedic medicine, sesame oil was used to treat various skin conditions and to promote wound healing. Ancient Egyptians also included sesame oil in their medicinal recipes for wound healing. Today, sesame oil is still used as a natural remedy for wounds and other skin problems [98]. Sesame oil has a variety of potential benefits for wound healing. Sesame oil's inherent anti-inflammatory properties help reduce swelling by inhibiting inflammatory mediators at the wound site, promoting faster tissue recovery. Its antibacterial characteristics combat microbial growth, preventing infection and creating a conducive environment for wound healing. It is also high in linoleic acid and vitamin E, both of which can help promote new skin growth and regeneration. Additionally, sesame oil has been shown to improve blood circulation and can provide extra nutrients to the area that can help speed up wound healing [99]. Sesame oil is a rich source of both antioxidants and fatty acids, which can help protect the wound from free radical damage, reduce inflammation, and promote healing. It also contains terpenes, which have antimicrobial properties that can protect the wound site from bacterial and fungal infections [100]. In addition, sesame oil is high in vitamin E, which helps to nourish and hydrate the skin, while also protecting it from environmental toxins and free radicals. Finally, sesame oil is easily absorbed into the skin, helping to speed up the healing process. Sesame oil can also be used as a natural analgesic for pain relief. Due to its anti-inflammatory properties, it can reduce swelling and discomfort at the wound site. Furthermore, sesame oil can help regulate the production of collagen, a vital protein for wound healing [101]. Additionally, sesame oil can help stimulate new tissue growth, reduce scarring, and promote the healing of wounds. Sesame oil has been shown to help reduce scarring and the formation of hypertrophic scars. It can also help improve skin elasticity and tone, prevent infection, reduce itching and irritation, and promote faster wound healing. Additionally, Natural antifungal qualities in sesame oil can help treat widespread fungi infections like jock itch and athlete's foot [102].

## 6 THE ROLE OF ALTERNATIVE MEDICINE IN WOUND HEALING

Homeopathy, herbal medicines, and other forms of alternative medicine can be quite effective in helping wounds heal. Pain and inflammation are frequently reduced using these treatments, promote circulation, and accelerate healing [103]. Acupuncture has been shown to boost the area's blood flow, which helps heal wounds faster. Herbal remedies can be used to reduce inflammation and improve the skin’s immune response, which can lead to faster healing [104]. Homeopathy has also been used for many years to help with the healing process. It is believed to help the body restore balance, reducing inflammation and promoting the circulation of energy. Each of these alternative medicines can be beneficial in wound healing, depending on the individual's condition and needs. In addition to traditional medical treatments, alternative medicine such as naturopathy, massage therapy, and Reiki can also play a role in wound healing [105]. Naturopathy focuses on the body's natural ability to heal itself using herbal remedies, dietary and lifestyle changes, and other natural methods. Massage therapy can help reduce inflammation, promote circulation, and reduce pain. Reiki is a form of energy healing that can help balance the body's energies and aid in the healing process. Each of these forms of alternative medicine can be beneficial when used in conjunction with conventional medical therapies to help encourage the healing of wounds. The use of alternative therapies in wound healing provides additional benefits that may not be achievable through traditional medical treatments alone [106]. For example, some alternative remedies can reduce infection risks and promote scar-free healing. Other forms of alternative medicine can help reduce pain and inflammation, as well as improve circulation to the affected area. Alternative therapies can also help boost the immune system, which can aid in fighting off any infection that may occur [107]. Ultimately, a combination of both traditional medical treatments and alternative therapies can provide the best results when treating a wound. In conclusion, alternative medicine can be a valuable addition to traditional medical treatments for wound healing. These therapies can help reduce inflammation and improve circulation, boost the immune system, reduce pain, and promote scar-free healing. Additionally, some forms of alternative medicine may provide additional benefits such as reducing infection risks. Ultimately, an individual's demands and symptoms should be taken into account when using alternative medicine for wound healing. And in most cases should be used in conjunction with traditional medical treatments [108].

### 6.1 Acupuncture Medicine

Acupuncture has been practiced as a healing method for thousands of years and is now recognized as an alternative therapy to facilitate injury recovery. It enhances the healing process by improving circulation, reducing inflammation, and promoting relaxation, which can alleviate pain and accelerate tissue repair [109]. Additionally, acupuncture stimulates the release of endorphins, natural pain relievers that support healing [110].

Studies also suggest that acupuncture increases collagen production, aiding in wound healing by reducing scarring and improving wound appearance [111]. By addressing multiple aspects of recovery—circulation, pain management, and tissue regeneration—acupuncture offers a holistic approach to enhancing the body’s natural healing processes [112].

### 6.2 Ayurveda

Ayurveda medicine has been used for centuries to treat wounds and promote healing. It is believed to be effective in reducing inflammation, promoting circulation, and stimulating the body's natural healing process. Herbal remedies, such as turmeric, neem, and aloe vera, are often used to treat wounds and promote healing [113]. Ayurvedic oils, such as sesame oil, are also used to massage the affected area and promote healing. Ayurvedic practitioners may also recommend lifestyle changes, such as avoiding certain foods, to help speed up the healing process [114]. Additionally, Ayurvedic practitioners may recommend specific yoga poses and breathing exercises to help reduce stress and promote healing. Ayurveda also emphasizes the importance of proper nutrition for wound healing. A balanced diet rich in fruits, vegetables, and whole grains might hasten the body's healing process [115]. Additionally, drinking plenty of water and avoiding processed foods can help the body heal more quickly. Ayurveda also recommends certain herbs and supplements to help promote wound healing. These include turmeric, neem, and aloe vera, as well as vitamins A, C, and E. Additionally, Ayurveda recommends certain Ayurvedic medicines, such as ashwagandha, to help reduce inflammation and promote healing.

### 6.3 Homeopathy

Homeopathic remedies are believed to stimulate the body's natural healing mechanisms, aiding in the reduction of pain, inflammation, and promoting the overall healing process. They are used for both acute and chronic wounds, including cuts, scrapes, burns, and ulcers. Additionally, homeopathic treatments can help manage infections, minimize scarring, and encourage tissue regeneration [116].

These remedies can be administered either orally or applied topically to the wound, depending on the condition being treated. By assisting the body’s natural healing processes, homeopathic remedies aim to reduce inflammation, alleviate pain, and accelerate recovery. Their versatility makes them a potential option for managing various types of wounds and associated complications, such as infection and scarring [117].

### 6.4 Naturopathy

Naturopathy plays a vital role in wound healing by offering natural remedies and treatments that reduce inflammation, enhance circulation, and alleviate pain. It also helps lower the risk of infection and accelerates the healing process. Naturopathic approaches such as herbal remedies, acupuncture, and massage contribute to these benefits by addressing inflammation and improving blood flow [118].

Beyond direct wound care, naturopathy supports the overall health of the patient, which is crucial for effective healing. Dietary guidance and lifestyle modifications provided by naturopathy can improve well-being, while emotional support and stress relief help reduce the risk of infection and promote faster recovery [119]. This holistic approach emphasizes the interconnectedness of physical, emotional, and overall health, making naturopathy a valuable complement to conventional wound care strategies.

### 6.5 Chinese or Oriental Medicine

Chinese or Oriental medicine has been practiced for centuries to promote wound healing and overall health. This holistic approach is based on the belief that the body is a network of energy pathways, and imbalances or blockages in these pathways can lead to illness and delayed healing [120]. To restore balance and support the body's natural healing processes, Chinese medicine employs a variety of techniques, including herbal remedies, acupuncture, massage, and dietary therapy.

Herbal treatments are commonly used to reduce inflammation, improve circulation, and enhance tissue repair. Acupuncture, another cornerstone of Chinese medicine, helps alleviate pain, reduce discomfort, and minimize the risk of infection. Massage and dietary adjustments further contribute to restoring the body's equilibrium and promoting overall well-being. These therapies are also effective in reducing scarring and improving the cosmetic appearance of wounds [121].

By combining these practices, Chinese medicine provides a comprehensive approach to wound healing, targeting both physical and energetic imbalances. Beyond wound care, these techniques contribute to improved health and vitality, emphasizing prevention and long-term wellness.

## 7 THE NOVEL TECHNOLOGIES AND STRATEGIES FOR WOUND HEALING

Novel strategies for wound healing is presented in Fig. (**3**).

### 7.1 Bioengineered Allogeneic Cellular Therapies

Bioengineered allogeneic cellular therapies are a promising new way to treat wounds and injuries. These therapies involve the use of living cells from a donor, which are engineered to produce beneficial proteins or other molecules when introduced into the patient’s body. The engineered cells can then help to promote the healing of the wound or injury, which can be particularly helpful in cases where traditional treatments are not effective [122]. Geographical data can help to identify areas where these therapies may be more beneficial due to the types of wounds and injuries that are most common in those areas. For example, data can be used to determine the prevalence of burns, diabetic ulcers, and pressure ulcers in different locations. This data can then be used to develop targeted strategies for providing these therapies in areas where they are most needed. Additionally, geographical data can be used to identify locations where there is a lack of access to these therapies and where additional resources may be needed to provide them. It can also be used to measure the effectiveness of the therapy in different locations and assess how it has been received [123].

A promising strategy for wound healing is the use of bioengineered allogeneic cellular treatments, particularly for chronic wounds that take more than 30 days to heal on their own. These treatments involve the use of bioengineered skin structures that can either act as a substrate or covering to speed up the healing of wounds or actively encourage wound healing by supplying growth factors and other materials. Autologous, allogeneic, or xenogeneic sources can be used to create bioengineered skin structures; however, allogeneic sources offer the advantage of being more widely accessible and reducing donor site morbidity. In addition, compared to allogeneic skin transplants taken from cadavers, bioengineered allogeneic skin structures can lower the likelihood of graft rejection and treatment failure. Clinical trials have been conducted on bioengineered allogeneic skin constructions, and the outcomes for chronic wounds and burn wounds are encouraging. As a result, bioengineered allogeneic cellular therapies can be viewed as a superior alternative to allopathic medicine for wound healing because they can promote tissue repair *in vivo* and restore functional skin tissue *in vitro*, as shown in Fig. (**4**) [124-126].

### 7.2 Stem Cell Therapy

Stem cell therapy, a form of regenerative medicine, uses stem cells to promote wound healing. Stem cells can differentiate into various cell types, aiding the body's natural healing process by encouraging tissue and blood vessel formation. This can reduce inflammation, scarring, and speed up recovery. Stem cell therapy also helps alleviate pain and discomfort by promoting healthy tissue growth and fighting infection, including bacterial, viral, fungal, and other pathogens. For example, it can combat bacteria like *S. aureus*, *E. coli*, *P. aeruginosa*, and *C. albicans* [127].

Stem cell therapy is particularly promising for chronic, non-healing wounds that do not respond to conventional treatments. These undifferentiated cells can self-renew and specialize into immune, skin, and blood vessel cells. Additionally, stem cells release cytokines and growth factors that regulate inflammation and promote angiogenesis, both crucial for healing [128-130].

Various types of stem cells, including embryonic stem cells (ESCs), induced pluripotent stem cells (iPSCs), and mesenchymal stromal cells (MSCs), have been investigated for wound healing. MSCs, being multipotent, easy to isolate, and low in immunogenicity, are the most commonly used. They can be sourced from bone marrow, adipose tissue, umbilical cord blood, and placenta. MSCs have been shown to accelerate granulation tissue formation, re-epithelialization, collagen synthesis, and vascularization in wounds [131, 132].

Despite its potential, stem cell therapy faces challenges, including limited cell survival, engraftment, and homing, as well as issues with cell heterogeneity, ethical concerns, and high costs. Approaches such as genetic engineering, exosome delivery, and biomaterial scaffolds have been developed to improve the efficacy of stem cells, enhancing their survival and function in the wound environment. Stem cell therapy offers advantages over traditional treatments by addressing the root causes of poor healing and restoring tissue function. However, further research is needed to optimize stem cell sources, delivery methods, and dosages for different wound types, as well as to evaluate the safety, efficacy, and long-term effects of this therapy (Fig. **5**) [128-130].

### 7.3 Xenograft Acellular Matrices Therapy

Xenograft acellular matrices, derived from animal tissues such as porcine or bovine dermis, are increasingly used in wound healing as scaffolds for tissue regeneration. These matrices provide temporary support, allowing the body's cells to migrate and proliferate, leading to new tissue formation and potentially accelerating healing while reducing scarring [133]. They can also deliver medications or growth factors to wounds, enhancing the healing process and offering a protective barrier against infection [133]. While still a relatively new technology, xenograft acellular matrices have shown promise in clinical trials and are expected to see wider use in the future [134].

Xenograft acellular matrix therapy (XAMT) involves applying a decellularized scaffold to the wound bed, providing structural support, growth factors, and a favorable environment for cell migration and proliferation. XAMT has been shown to improve tissue regeneration, wound closure, revascularization, and re-epithelialization in various wound types, including burns, ulcers, and surgical defects as shown in Fig. (**6**) [135-138]. Compared to allopathic treatments, XAMT is less likely to cause allergic reactions, drug resistance, or persistent inflammation, making it an attractive alternative, particularly for complex, hard-to-heal wounds. However, further research is necessary to refine its materials, processing methods, and clinical applications [139].

### 7.4 3D Printed Stem Cells Therapy

3D-printed stem cells hold immense potential for advancing wound healing. Stem cells possess the unique abilities of self-renewal and differentiation into various cell types, including those crucial for tissue repair and regeneration. Using 3D printing technology, scaffolds can be engineered to house stem cells, promoting new tissue growth and facilitating the healing process [140].

Moreover, 3D-printed stem cells enable the development of personalized treatments tailored to individual patient needs. This approach reduces the risk of complications and improves overall treatment outcomes. Additionally, these cells can be integrated into tissue-engineered products, replacing damaged or missing tissues [141]. The technology for 3D printing stem cells involves specialized bioprinters, which are revolutionizing regenerative medicine by creating personalized therapies and advanced tissue-engineered products. In the future, 3D-printed stem cells could contribute to developing complex organs and tissues, addressing various diseases and conditions [142].

Traditional allopathic wound care often relies on medications and surgical interventions, which may lead to side effects such as infections, inflammation, scarring, and chronic pain. Furthermore, allopathic treatments cannot fully restore complex skin structures and functions, such as hair follicles, sweat glands, and pigmentation. In contrast, 3D-printed stem cell therapy delivers living cells directly to the wound site, harnessing the body’s natural healing processes. Stem cells can differentiate into various cell types and release cytokines and growth factors that modulate immune responses and aid tissue regeneration. Using 3D printing, custom scaffolds with precise control over mechanical properties, size, shape, and porosity can be fabricated. These scaffolds mimic the extracellular matrix of the skin, providing an optimal environment for stem cell attachment, proliferation, and differentiation.

Numerous studies have demonstrated the advantages of 3D-printed stem cell therapy over allopathic methods. For instance, in a rat model of full-thickness excisional wounds, medical-grade polycaprolactone (mPCL) dressings seeded with human gingival tissue multipotent mesenchymal stem/stromal cells (hGMSCs) significantly enhanced skin regeneration while minimizing scarring. These dressings improved granulation and re-epithelialization, reduced wound contracture, and promoted physiological wound closure while limiting scar formation.

Additionally, 3D bioprinting allows for the incorporation of antibacterial agents such as silver nanoparticles, antibiotics, and bacteriophages into scaffolds. This enhances the antibacterial properties of stem cell therapy, preventing infections and improving wound healing outcomes. Researchers have emphasized the potential and challenges of 3D bioprinting for delivering external stem cells and antibacterial agents to wounds, underscoring its transformative applications.

3D-printed stem cell therapy offers superior advantages over traditional allopathic medicine for wound healing by delivering living cells capable of rebuilding skin tissue and combating infections. The ability to fabricate customized scaffolds that support stem cell survival and functionality enhances the efficacy of treatment. With further advancements, 3D-printed stem cell therapy holds the potential to revolutionize wound care and improve the quality of life for patients suffering from severe skin injuries (Fig. **7**) [143, 144].

### 7.5 Growth Factors Therapy

Growth factors therapy utilizes naturally occurring proteins to promote wound healing, focusing on critical processes such as angiogenesis, cell growth, collagen formation, and inflammation reduction. These proteins help stimulate the development of new blood vessels and accelerate tissue regeneration, essential for wound healing. Chronic wounds, including diabetic foot ulcers (DFUs), venous leg ulcers, and pressure ulcers, benefit from this therapy, and it is also applicable to acute wounds such as surgical incisions [145].

This therapy not only aids in reducing pain, discomfort, and scarring but also helps prevent infection, improving the overall wound appearance. It is generally safe, well-tolerated, and can be combined with other treatments to enhance wound healing [146]. Growth factor delivery devices, commonly used for administering these proteins, help ensure the controlled application of the growth factors to the wound site.

Key growth factors used in therapy include transforming growth factor (TGF), epidermal growth factor (EGF), and platelet-derived growth factor (PDGF). These proteins play a significant role in stimulating collagen production, a vital element for wound healing [147]. Over time, growth factor therapy may be integrated with other approaches, such as stem cell therapy, to further accelerate wound healing processes.

Growth factor therapy offers several advantages over traditional allopathic treatments, including the promotion of angiogenesis, granulation tissue formation, epithelialization, and collagen synthesis. It also aids in the reduction of infection rates, inflammation, and scarring, and accelerates the overall healing process. This non-surgical treatment can be applied to a wide range of wound types, such as burns, diabetic foot ulcers, chronic ulcers, and surgical wounds [148, 149].

Several growth factors, including TGF, fibroblast growth factor (FGF), insulin-like growth factor (IGF), keratinocyte growth factor (KGF), PDGF, and vascular endothelial growth factor (VEGF), have been clinically used to promote wound healing. These factors can be delivered *via* injections or topically, either alone or in combination with other treatments, such as biomaterials, oxygen therapy, or gene therapy [148, 149].

Numerous studies have validated the effectiveness and safety of growth factor therapy in wound healing. For example, PDGF-BB significantly improved the healing rate of DFUs compared to a placebo in a randomized clinical trial in the United States. Similarly, the combination of topical oxygen therapy and bFGF has shown promise in healing deep partial-thickness burns. Clinical guidelines have also recommended the use of EGF as an adjuvant therapy for chronic wounds resistant to conventional treatments.

Overall, growth factor therapy offers several advantages over allopathic medicine, making it a promising approach for accelerating wound healing. It enhances the body’s natural healing processes, facilitating better recovery for various types of wounds, and is supported by a growing body of research, demonstrating its efficacy in treating chronic and acute wounds, as shown in Fig. (**8**) [150-152].

### 7.6 Collagen-based Wound Healing Therapy

Collagen-based wound healing therapy utilizes collagen, a natural protein essential for tissue repair, to promote wound healing (Fig. **9**). Collagen-based products can be applied topically or injected into the wound site to stimulate the body's natural healing process. These products help reduce inflammation, support tissue regeneration, minimize scarring, and alleviate pain and discomfort [153].

This therapy is considered safe and effective and can be combined with other treatments like antibiotics and dressings. Collagen used in these therapies is often derived from animal sources, such as bovine or porcine tissues. However, human-derived collagen, obtained from donated tissues or a patient’s own body, is known to be more effective in promoting wound healing due to its higher compatibility and reduced risk of immune reactions [154].

Collagen-based wound healing therapy involves syringes, needles, and topical applicators. This therapy is adaptable to various wound types, including chronic, acute, and burn wounds. Collagen-based products are generally well- tolerated, pose minimal risk of immunological reactions, and can attract beneficial cells to the wound site. They work by accelerating the natural healing process without relying on artificial chemicals that may cause complications.

Research supports the use of collagen-based products for a wide range of wound types. For example, studies have shown that collagen sponge dressings significantly improve wound healing outcomes for diabetic foot ulcers compared to traditional dressings [155, 156]. Ongoing research continues to explore innovative ways to harness collagen’s potential in wound healing, making this therapy a promising alternative to conventional allopathic treatments [157].

### 7.7 Fluorescence Points of Care Imaging Therapy

Fluorescence point-of-care imaging plays a critical role in wound healing by providing real-time information about the wound environment. This technology helps monitor healing progress, detect areas of infection, and guide treatment decisions. It can identify the presence of bacteria, fungi, and other microorganisms, aiding in the selection of appropriate antibiotics or treatments [158]. Additionally, fluorescence imaging detects oxygen and nutrient levels in wounds, optimizing the healing process and enabling timely interventions.

Fluorescence imaging can also assess collagen quality, including types I, II, and III, providing insights into tissue regeneration and guiding treatment strategies. It can detect inflammatory cells, helping identify areas of infection or inflammation requiring targeted interventions [159]. Instruments such as the Cystoviral Hyperspectral Imaging System are commonly used for fluorescence imaging, offering real- time images to support rapid assessment and treatment.

In the future, fluorescence imaging may detect biomarkers and bioactive molecules, such as growth factors or cytokines, to further enhance wound healing. Studies have shown its effectiveness in improving outcomes for diabetic foot ulcers and other hard-to-heal wounds. For instance, a UK NHS study (2018-2020) reported a 23% increase in diabetic foot ulcer healing rates within 12 weeks when MolecuLight fluorescence imaging was used. Another study found that targeted debridement based on fluorescence imaging reduced wound area by an average of 27.7% per week in lower extremity wounds.

Compared to traditional wound care techniques like visual examination or microbiological sampling, fluorescence imaging provides real-time, non-invasive, and reliable information at the bedside. This enables precise interventions, improves treatment outcomes, and reduces the risk of infection and complications. Unlike some allopathic approaches, fluorescence imaging minimizes adverse effects such as toxicity or resistance and addresses underlying factors like poor blood circulation and inadequate nutrition.

Fluorescence point-of-care imaging therapy holds promise as a transformative tool for wound care, offering significant advantages over conventional methods. However, further research is needed to evaluate its long-term benefits, cost-effectiveness, and integration into standard wound care protocols [160-163].

### 7.8 Bacteria Killing Laser Therapy

Bacteria-killing laser therapy is an innovative approach to wound healing that targets and destroys bacteria responsible for infections (Fig. **10**). This therapy reduces the risk of infection, promotes faster healing, minimizes inflammation and pain, and helps reduce scarring, thereby improving the overall appearance of wounds. Unlike collagen, which is essential for tissue repair, bacteria-killing laser therapy is specifically designed to target bacterial cells without affecting collagen [164].

Recent advances have shown that bacteria-killing lasers can eliminate multidrug-resistant bacteria and their spores without harming human cells. The laser works by vibrating and breaking bacterial protein structures, causing biochemical disruption and eventual bacterial death. Combining this therapy with other agents, such as copper nanoparticles or photosynthetic bacteria, can enhance its efficacy. For instance, hydrogels containing copper nanoparticles can convert localized heat from laser irradiation into copper ions with antibacterial and tissue-proliferative properties. Similarly, bioactive hydrogels with photosynthetic bacteria can create an aerobic environment conducive to wound healing by generating oxygen under light exposure [165].

This therapy offers several advantages over traditional allopathic wound care. It effectively targets a wide range of bacteria, including antibiotic-resistant strains, promotes collagen synthesis crucial for tissue repair, and reduces inflammation and pain by modulating immune and nervous system responses. Moreover, it is a non-invasive, safe, and straightforward method with minimal side effects.

Looking ahead, bacteria-killing laser therapy holds potential for broader applications, such as treating skin conditions, infections, and even cancer. Ongoing research focuses on refining this technology, exploring its ability to stimulate tissue regeneration, and evaluating its long-term effects. As technology advances, bacteria-killing lasers are expected to play a larger role in medical treatments, accelerating tissue regeneration and enhancing wound care outcomes [166-168].

### 7.9 Topical Oxygen Therapy

Topical oxygen therapy (TOT) has been used for wound healing since the early 1900s, initially to treat burns and later expanded to manage chronic wounds, such as diabetic foot ulcers (DFUs), pressure ulcers, and venous leg ulcers [169]. In recent years, TOT has gained popularity as a non-invasive wound care method that delivers oxygen directly to the wound site to enhance healing.

TOT increases oxygen levels in the wound bed, promoting angiogenesis, reducing inflammation, and inhibiting bacterial growth. It is particularly effective against anaerobic bacteria, which thrive in low-oxygen environments, and stimulates collagen production, a crucial protein for tissue repair and wound closure [170, 171]. TOT is typically administered using an oxygen concentrator, a device that extracts and concentrates oxygen from ambient air.

Research highlights its effectiveness in managing chronic wounds. A randomized controlled trial (RCT) by Serena *et al*., and Tang *et al*., in 30 DFU patients demonstrated that TOT significantly reduced wound size and accelerated healing compared to standard care (SOC) over 12 weeks, with no reported adverse effects. Similarly, a clinical trial with 100 DFU patients found that TOT combined with SOC outperformed SOC alone in wound closure rate (50% *vs.* 32%), wound size reduction (72% *vs.* 51%), and healing time (63 *vs.* 84 days). Adverse events were comparable between groups and mainly infection-related [172, 173].

Another systematic review by Edge and Frey evaluated the clinical efficacy and cost-effectiveness of continuously dispersed oxygen therapy (CDO), a form of TOT that delivers oxygen through a wet dressing. Across six studies with 1,029 patients, CDO improved wound healing outcomes compared to SOC or placebo, although evidence quality was low to moderate. One study also reported CDO to be cost-effective, but no guidelines recommending its routine use were identified [174].

Future directions for TOT include its combination with hyperbaric oxygen therapy or other advanced wound care treatments to further optimize healing. By enhancing oxygen delivery, TOT supports the natural healing process, reduces the need for debridement and antibiotics, and accelerates recovery in patients with chronic wounds (Table **2**) [172, 173].

### 7.10 Shock Wave Therapy

Shock wave therapy is a non-invasive medical technique used to stimulate tissue regeneration and enhance wound healing. It promotes blood vessel formation and increases cell growth factors, aiding tissue repair. Additionally, it helps reduce swelling, pain, and inflammation while improving cellular oxygenation [176].

Originally developed in the mid-1980s for orthopedic treatments and chronic tendon injuries, shock wave therapy has since been studied for its potential in treating chronic wounds, including diabetic foot ulcers, pressure ulcers, and leg ulcers. It has also been used for burn injuries, radiation wounds, scars, and post-surgical wounds [176].

The therapy is typically administered using an extracorporeal shock wave lithotripter (ESWL), which delivers focused acoustic waves to the wound site. This process promotes collagen production, a crucial component of connective tissue that provides skin strength and elasticity. It also enhances blood vessel formation and stimulates the growth of new tissue [177].

Future research on shock wave therapy includes exploring its efficacy in reducing healing times for acute wounds and its integration with other treatments, such as hyperbaric oxygen therapy. Its potential to further improve outcomes for chronic wounds, particularly in diabetic patients, holds promise for expanding its therapeutic applications.

### 7.11 Thermal Imaging Therapy

Thermal imaging therapy has been utilized for centuries to aid wound healing, with historical references to heated stones and hot compresses in ancient medical texts from India, China, and Egypt. Modern technological advances have refined this practice, enabling precise and controlled heat application to enhance wound healing [178]. Today, thermal imaging therapy is widely used in hospitals, clinics, and even private settings to accelerate wound recovery.

This therapy works by applying localized heat to the wound site, stimulating natural healing processes in the skin and underlying tissues. Increased blood flow from the heat brings oxygen and nutrients to the area, promoting faster tissue repair. It also reduces inflammation, pain, and edema while minimizing scarring and biofilm formation, which can cause infections [179]. Thermal imaging therapy is particularly beneficial for chronic or difficult-to-treat wounds due to its ability to create an environment conducive to faster healing.

Thermal imaging devices, the primary instruments used for this therapy, measure wound site temperature and help determine when heat application is necessary [180]. These devices can also track healing progress by monitoring temperature variations over time. Studies have shown that thermal imaging therapy can predict wound healing outcomes more effectively than traditional methods like digital planimetry or wound tracings, especially for venous leg ulcers (VLUs) [181, 182].

VLUs, which affect 1-2% of the population, are chronic wounds that often lead to pain and reduced quality of life. Thermal imaging therapy has been shown to enhance wound management and lower infection risk in such cases. Future advancements in thermal imaging technologies, such as infrared and ultrasound-based systems, may offer even more accurate and targeted applications. As understanding of the role of heat in wound healing grows, new and improved treatments are expected to emerge [183].

### 7.12 Telehealth Services Therapy

Telehealth services can play an important role in wound healing by providing remote access to healthcare professionals who can provide advice and guidance on wound care see Fig. (**11**). This can include education on proper wound care techniques, monitoring the wound for signs of infection, and providing advice on managing pain and discomfort [184]. Telehealth services can also provide access to specialized wound care services, such as wound debridement, compression therapy, and wound dressings. Telehealth services can also provide access to specialized wound care services, such as wound debridement, compression therapy, and wound dressings.

Additionally, telehealth services can provide access to wound care specialists who can provide advice on the best course of treatment for a particular wound [185]. Finally, telehealth services can provide access to wound care resources, such as educational materials and support groups, which can help patients better understand their wounds and how to manage them. One of the most commonly used instruments in telehealth services for wound healing is the Wound Care Assessment Tool (WCAT). This tool is used to assess the severity of a wound and to provide guidance on the best course of treatment. In the future, telehealth services may also incorporate more advanced technologies, such as artificial intelligence and machine learning, to provide more personalized and accurate wound care advice [186].

## 8 NANOFIBERS AND NANOFIBROUS MATRICES STRATEGIES

The use of nanofibers and nanofibers matrices for wound healing has a long and varied history. Ancient civilizations used herbs, oils, and animal hides as wound dressings to promote wound healing, and in the 20^th^ century, researchers developed hydrogel materials for this purpose. In the 21^st^ century, advances in nanotechnology have enabled the development of nanofiber-based materials with increased surface area, porosity, and mechanical strength. These materials can provide a more favorable environment for effective cell adhesion, migration, and proliferation compared to traditional wound dressings and hydrogels [188]. Nanofibers matrices have been shown to provide faster healing and enhanced wound closure compared to other treatments. As a result, they have become increasingly popular in wound care. Numerous research studies have demonstrated the successful application of nanofibers matrices for a variety of wound healing applications, such as burn wounds, diabetic foot ulcers, hypertrophic scars, and chronic non-healing wounds. In addition, nanofibers matrices can be further modified to include various therapeutic and regenerative components, such as antimicrobial agents and growth factors, which can provide additional benefits to help promote wound healing [189]. Nanofibers and nanofibers matrices have been increasingly studied as potential materials for wound healing. They are capable of mimicking the natural extracellular matrix and allowing for the delivery of drugs and biologics in the right place and time while providing a suitable microenvironment to foster the growth and differentiation of cells. Nanofibers can also be used as a scaffold to facilitate tissue regeneration by providing mechanical support and promoting cell migration, proliferation, and organization [190]. In addition, they can be functionalized with active molecules, such as bioactive peptides, to further enhance their tissue regenerative and healing effects. The future of nanofibers and nanofiber matrices in wound healing is promising, with researchers actively exploring new applications and materials. In particular, the use of biodegradable nanofiber matrices that can degrade over time is becoming increasingly popular, as this allows for more controlled delivery of therapeutic agents and improved tissue regeneration.

### 8.1 Classification of Nanofibers

Classification of nanofibers are presented in the Table **3**.

## CONCLUSION

Significant progress has been made in understanding wound healing mechanisms and developing innovative therapies to address chronic, non-healing wounds. However, achieving complete skin regeneration remains a substantial challenge due to the multifaceted nature of wound healing, which involves complex cellular and molecular pathways. Advanced strategies such as nanofibers, stem cell therapy, 3D bioprinting, and growth factor delivery have the demonstrated potential to accelerate healing and improve outcomes. Yet, these approaches often face limitations, including high costs, technical complexities, and variable efficacy across wound types. This review underscores the importance of combining traditional and advanced therapeutic methods to enhance treatment efficacy. Addressing existing gaps, such as the integration of patient-specific therapies and the exploration of nutritional impacts on wound healing, is crucial for advancing clinical practices. Future research should focus on optimizing novel technologies, ensuring affordability, and tailoring interventions to individual patient needs. By leveraging interdisciplinary approaches and overcoming current barriers, the field of wound care can progress toward achieving effective and comprehensive healing for complex wounds.

## POTENTIAL IMPACT AND FUTURE DIRECTIONS

The findings presented in this review have significant implications for advancing the treatment of non-healing wounds, a critical area in wound management. One of the major challenges in this domain is the association between chronic wounds and osteomyelitis, a severe bone infection that complicates wound healing. Highlighting the importance of timely diagnosis and effective treatment of osteomyelitis could lead to better clinical outcomes. Furthermore, the role of diet in wound healing remains an underexplored avenue that holds potential for groundbreaking insights. Investigating specific dietary components and their impact on the molecular and cellular mechanisms of wound repair could inspire future therapeutic strategies. While this review provides a comprehensive overview of both traditional and advanced therapies, it also acknowledges the limitations in our current understanding of wound-healing mechanisms. Addressing these gaps through interdisciplinary research will be pivotal in developing innovative, patient-specific approaches to improve chronic wound management.

## Figures and Tables

**Fig. (1):**
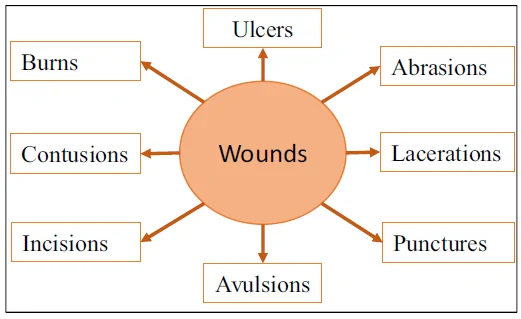
Types of wounds.

**Fig. (2):**
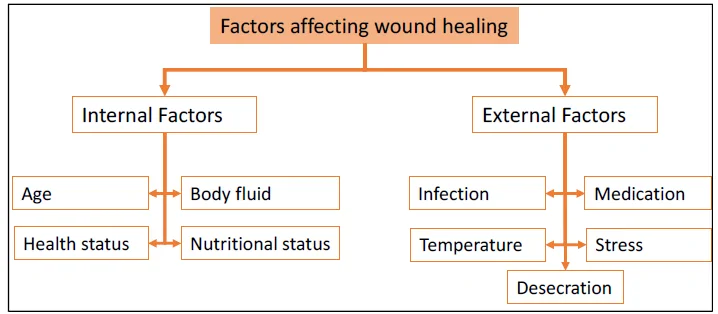
Factors affecting wound healing.

**Fig. (3):**
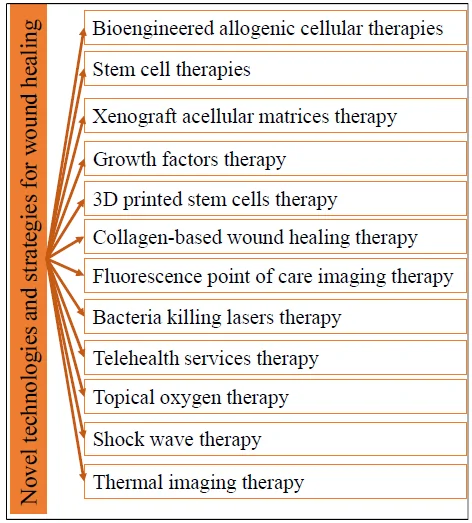
Novel strategies for wound healing.

**Fig. (4):**
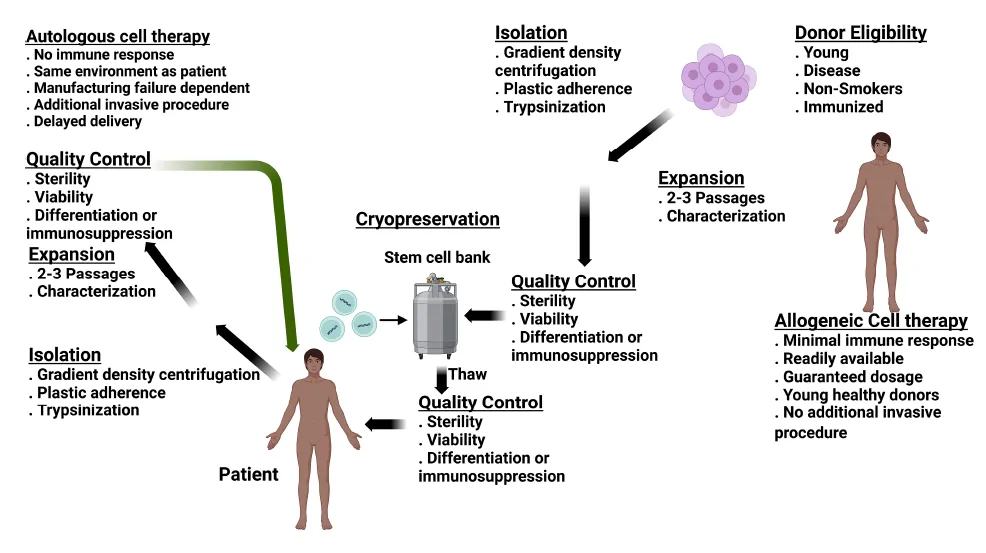
Bioengineered allogeneic cellular therapies.

**Fig. (5):**
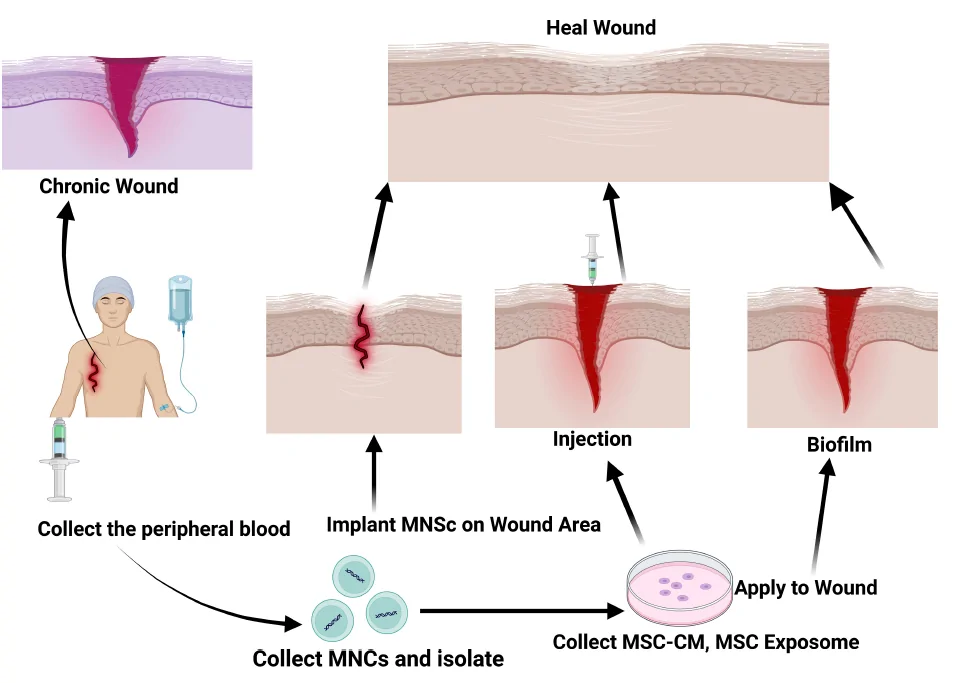
Stem cell therapy.

**Fig. (6):**
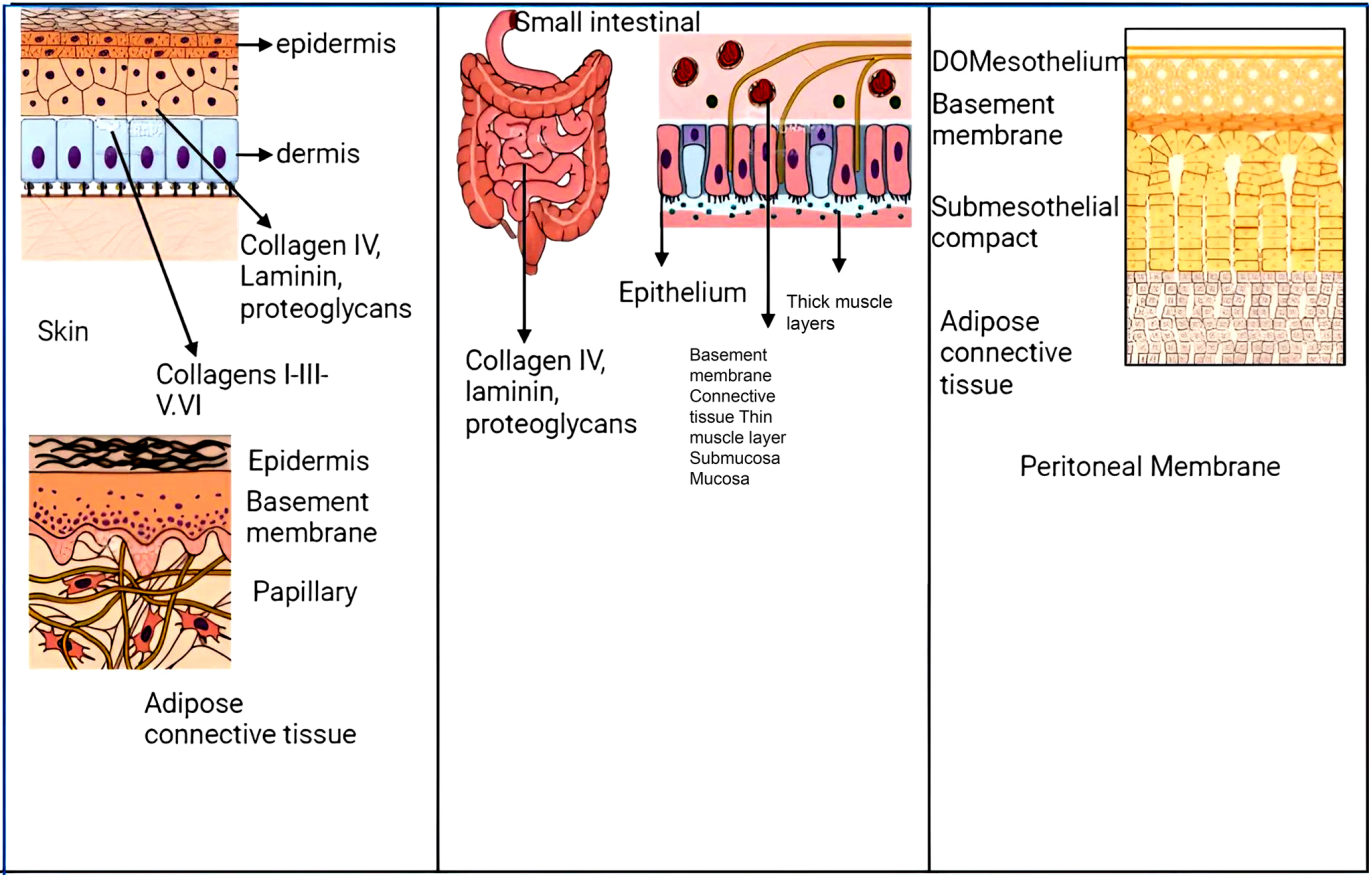
Xenograft acellular matrices therapy.

**Fig. (7):**
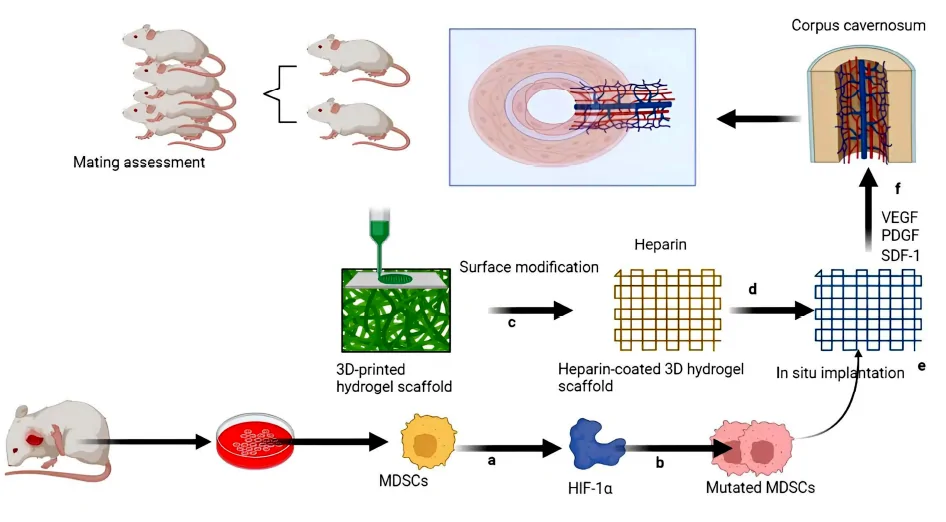
3D printed steam cell therapy.

**Fig. (8):**
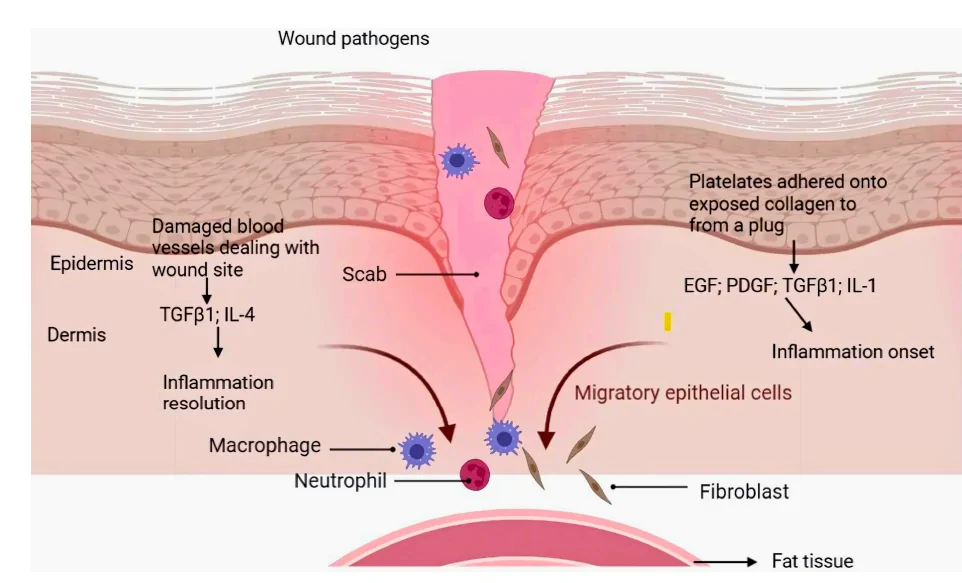
Growth factors therapy.

**Fig. (9):**
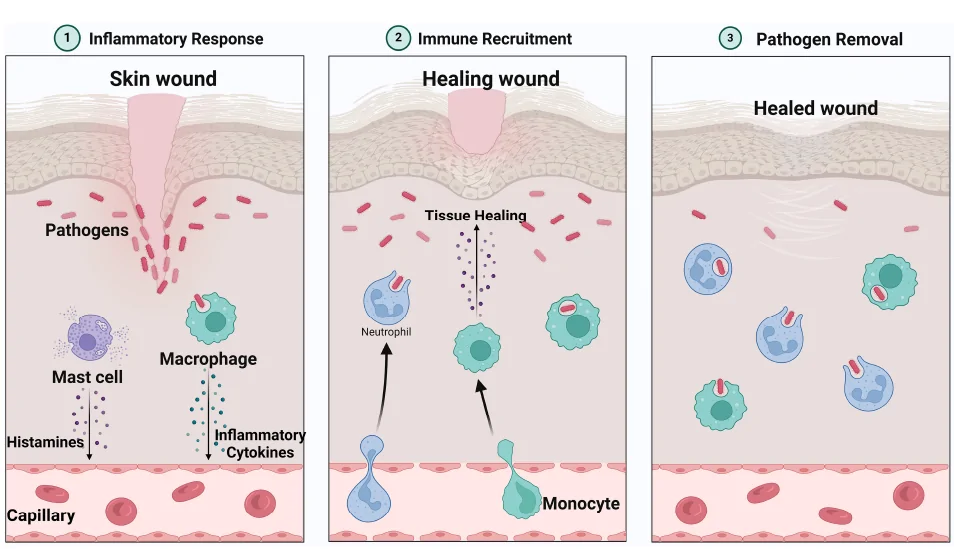
Collagen based wound healing therapy.

**Fig. (10):**
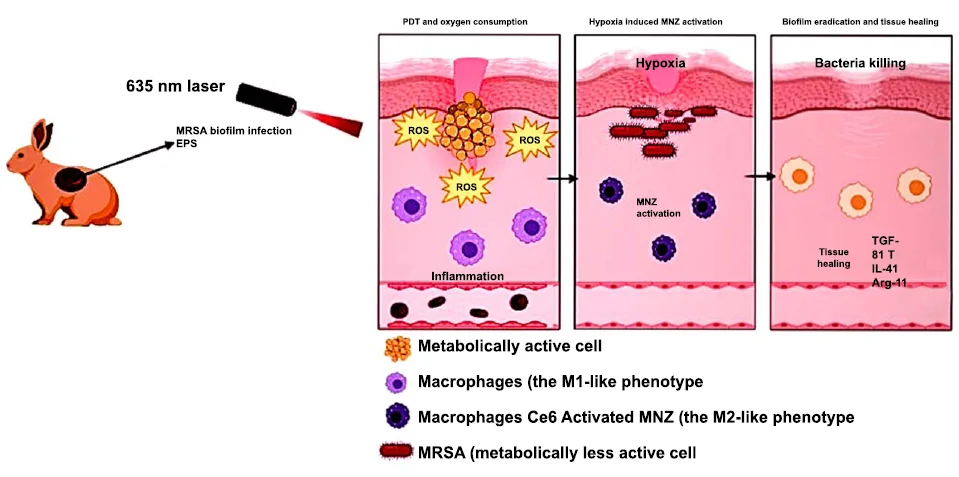
Bacteria killing laser therapy.

**Fig. (11):**
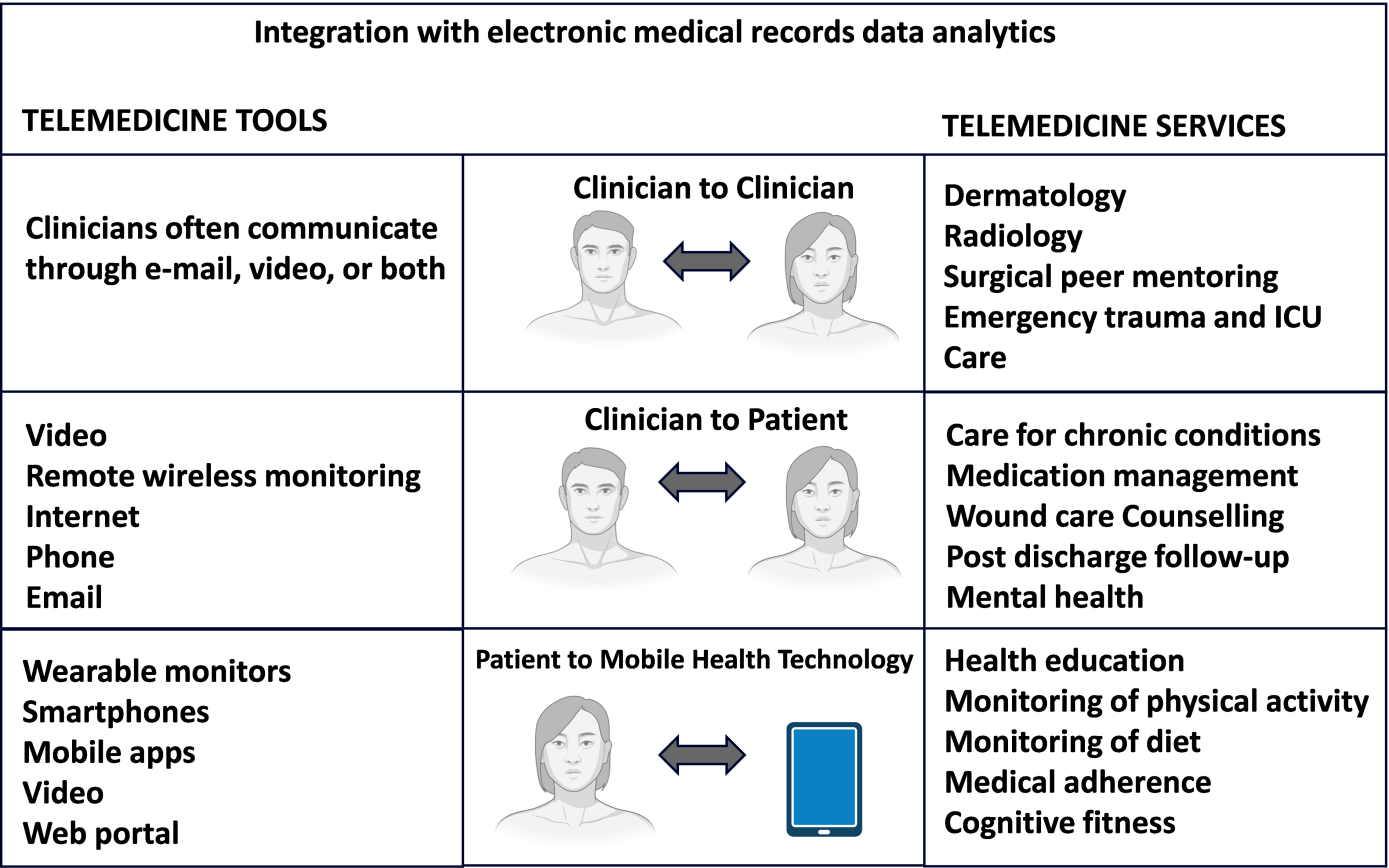
Tele services therapy [187].

**Table 1:** Traditional medicines for wound healing.

**S.No.**	**Plants**	**MOA**	**Phases**	**Wounds**	**Formulations**	**References**
1.	Aloe Vera	Glucomannan substance helps cellular regeneration in the body and produces collagen.	Inflammation and preventing ulcer	First and second burn wound	Nanocomposite, nanosuspension, nanofibrous, and silk-based	[46-50]
2.	Honey	Peroxide generation, antioxidant activity, proinflammatory cytokines, MMP9, TGFβ, hygroscopic effect.	Inflammatory phase, proliferative phase, remodeling phase	Infected wound	Honey-based hydrogel, micro sponge-loaded hydrogel, biodegradable hydrogel sponge, hyaluronic acid-based hydrogel, alcohol-chitosan hydrogel	[51-55]
3.	Turmeric	Permit immune and other cells necessary for healing and modifying wounds to migrate. Any protracted inflammatory pathways are blocked by turmeric.	Protective inflammatory phase	Inflammatory wound	Polyherbal formulation, transdermal drug delivery, hydrogels, curcumin-loaded nano preparations, polymeric micelles.	[56-60]
4.	Tea tree oil	Investigations were done on the effectiveness of tea tree oil and three of its constituents, 1,8-cineole, terpinen-4-ol, and -terpineol, against *Staphylococcus aureus* ATCC 9144.	Inflammation phase	Inflammatory wound	Nanostructured lipid carriers, oil-loaded chitosan topical preparations, oil solution, nano capsules, nano emulsion	[61-65]
5.	Coconut oil	Cell organ retention, fibroblast proliferation, and neovascularization are all considerably enhanced by this process.	Inflammation phase	Burn wound	Nano emulsion, a classical Siddha formulation, incorporated gelatin-based film	[66-68]
6.	Sesame oil	Due to its antioxidant activity, sesame oil extract aids in the prevention of oxidative damage and speeds up wound healing.	Inflammation phase	Second degree burn wound	Ozonated oil, nano emulsion gel, polyherbal,	[69-71]

**Table 2:** Topical oxygen therapy.

**Modality**	**Continuous Diffusion of Oxygen Therapy (CDO)**	**Topical Oxygen Therapy^A^ (TO)**	**Hyperbaric Oxygen Therapy (HBO)**
Treatment at home	YES	Yes^B^	No (travel to the facility)
Wearable, 24/7 patient mobility	YES	No (patient immobile 90 min)	No (patient immobile 90 min)
Complete system with dressings	YES	No	No
Direct wound oxygenation	YES	YES^C^	No (min TCOM req'd)
Continuous wound oxygenation	YES	No (patient immobile 90 min)	No (only 90 min/day)
Absence of fire Hazard	YES	No (high-flow oxygen)	No (high flow oxygen)
Battery operated	YES	No (uses 600-1300W, heat)	No (high power)

**Note: **A -Topical Hyperbaric Oxygen Therapy (THOT) or Topical Wound Oxygen (TWO) therapy, B -Treated at home, yet patient immobile for an hour and a half during treatment, C - Treatment typically treats portion of limb, not just wound: limb may be constricted [175].

**Table 3:** Classification of nanofibers.

**S.No.**	**Based on**	**Types**	**Formulations**	**References**
1.	Raw material	Organic, inorganic, and carbon and composite fibers.	Organic and inorganic electro spun nanofibers, effect of microstructures of carbon nanoproducts grown on carbon fibers on the interfacial properties of epoxy composites.	[2, 191]
2.	Structure	Nonporous, mesoporous, hollow, and core-shell fibers.	Core/Shell nanoparticles: classes, properties, synthesis mechanisms, characterization, and applications, design of porous, core-shell, and hollow nanofibers.	[192, 193]
3.	Fabrication method	Electrospinning, melt-blowing, and template-assisted methods.	Electrospinning and electrospun nanofibers: methods, materials, and applications, melt-blown and electrospun drug-loaded polymer fiber mats for dissolution enhancement: a comparative study.	[194]
4.	Application	Filtration, tissue engineering, drug delivery, energy storage, and sensing.	Tissue engineering in drug delivery, sterilizing filtration of liposome and related lipid-containing solutions: enhancing successful filter qualification.	[195, 196]
5.	Diameter	Ultrafine, submicron, and nanofibers.	Synthesis of ultrafine polymer nanofibers.	[197]
6.	Morphology	Straight, branched, and tangled fibers.	Different methods for nanofiber design and fabrication.	[198]
7.	Surface properties	Hydrophilic, hydrophobic, and amphiphilic fibers.	Synthesis of hydrophobic and hydrophilic graphitic carbon nanofiber polymer brushes.	[199]
8.	Electrical properties	Conductive, semi-conductive, and insulating fibers.	Magnetic profiling of conducting, semi-conducting, and insulating nanoparticles and their nanofluids possessing potential transformer application.	[200]
9.	Mechanical properties	Flexible, elastic, and rigid fibers	Flexible, fatigue resistant, and heat-insulated nanofiber-assembled polyimide aerogels with multifunctionality.	[201]
10.	Chemical properties	Reactive, inert, and biodegradable fibers.	Bioactive thermoresponsive polyblend nanofiber formulations for wound healing.	[202]
11.	Optical properties	Transparent, translucent, and opaque fibers.	Nanofiber-based transparent film with controllable optical transparency adjustment function for versatile bionic applications.	[203]
12.	Thermal properties	Thermally conductive, thermally insulating, and thermally resistant fibers.	Lightweight, flexible, thermally-stable, and thermally-insulating aerogels derived from cotton nanofibrillated cellulose.	[204, 205]
13.	Magnetic properties	Ferromagnetic, paramagnetic, and diamagnetic fibers.	The comparison between superparamagnetic and ferromagnetic iron oxide nanoparticles for cancer nanotherapy in the magnetic resonance system.	[206]
14.	Shape	Cylindrical, conical, and helical fibers.	Controllable growth of conical and cylindrical tio2-carbon core-shell nanofiber arrays and morphologically dependent electrochemical properties.	[207]
15.	Surface chemistry	Functionalized, non-functionalized, and modified fibers.	Surface functionalization of nanofibers: the multifaceted approach for advanced biomedical applications.	[208]

## LIST OF ABBREVIATIONS

DFUs: Diabetic Foot Ulcers
EGF: Epidermal Growth Factor
MCTs: Medium-chain Triglycerides
mPCL: Medical-grade Polycaprolactone
PDGF: Platelet-derived Growth Factor
TOT: Topical Oxygen Therapy
TGF: Transforming Growth Factor
